# Determinants of Success and Failure in Prehospital Endotracheal Intubation

**DOI:** 10.5811/westjem.2016.6.29969

**Published:** 2016-07-26

**Authors:** Lucas A. Myers, Charles G. Gallet, Logan J. Kolb, Christine M. Lohse, Christopher S. Russi

**Affiliations:** * Mayo Clinic, Gold Cross, Rochester, Minnesota; † Mayo Clinic, Division of Biomedical Statistics and Informatics, Rochester, Minnesota; ‡ Mayo Clinic, Department of Emergency Medicine, Rochester, Minnesota

## Abstract

**Introduction:**

This study aimed to identify factors associated with successful endotracheal intubation (ETI) by a multisite emergency medical services (EMS) agency.

**Methods:**

We collected data from the electronic prehospital record for all ETI attempts made from January through May 2010 by paramedics and other EMS crew members at a single multistate agency. If documentation was incomplete, the study team contacted the paramedic. Paramedics use the current National Association of EMS Physicians definition of an ETI attempt (laryngoscope blade entering the mouth). We analyzed patient and EMS factors affecting ETI.

**Results:**

During 12,527 emergent ambulance responses, 200 intubation attempts were made in 150 patients. Intubation was successful in 113 (75%). A crew with paramedics was more than three times as likely to achieve successful intubation as a paramedic/emergency medical technician-Basic crew (odds ratio [OR], 3.30; p=0.03). A small tube (≤7.0 inches) was associated with a more than 4-fold increased likelihood of successful ETI compared with a large tube (≥7.5 inches) (OR, 4.25; p=0.01). After adjustment for these features, compared with little or no view of the glottis, a partial or entire view of the glottis was associated with a nearly 13-fold (OR, 12.98; p=0.001) and a nearly 40-fold (OR, 39.78; p<0.001) increased likelihood of successful intubation, respectively.

**Conclusion:**

Successful ETI was more likely to be accomplished when a paramedic was partnered with another paramedic, when some or all of the glottis was visible and when a smaller endotracheal tube was used.

## INTRODUCTION

Endotracheal intubation (ETI) performance by emergency medical services (EMS) personnel remains a heavily examined and debated issue for medical directors and prehospital care providers. Research on success rates in adults has demonstrated ranges from 77.2% to 98.5%.^1^–^4^ Unfortunately, opportunities for clinical intubation are infrequent.^5^ EMS educational programs have highlighted the need for greater frequency of ETI performance through clinical opportunities such as the operating suite.^6^ Given the relatively few opportunities for practicing the procedure in some EMS systems, detailed patient selection and guideline criteria aimed at limiting difficult intubation attempts may increase the relative proportion of success.

We sought to determine prehospital ETI success rates and to identify the factors associated with success and failure in a single EMS system serving patients in both rural and semi-urban settings.

## METHODS

This study was approved by our institutional review board. In this prospective, observational study of a single, multisite ambulance provider, we analyzed prehospital patient care reports for adult and pediatric patients who underwent attempted ETI at any time for any cause from January through May 2010. For electronic medical record entry, this service uses the EMSPro (Zoll Medical Corp), which allows changes to be made to the electronic documentation record.

Documentation is made in the medical record by a paramedic whenever an intubation is performed. All EMS staff, systemwide, were trained before the beginning of data collection and were expected to complete necessary documentation points. Paramedics used the current NAEMSP (National Association of EMS Physicians) definition of an ETI attempt (laryngoscope blade entering the mouth).^7^ The scope of practice in advanced airway management allows for only paramedic-trained staff to perform intubation. Agency protocol requires the gum elastic bougie to be placed through the cords during each attempt, with the partner placing the endotracheal tube over the gum elastic bougie, while the intubator continues to directly visualize the cords (no video laryngoscopy was used). There was evidence of some deviation from this practice in this sample. Intubation success was self-reported by the paramedic in the electronic medical record.

After reviewing the captured baseline data elements in the Zoll electronic medical record, the study team added airway-specific variables to the EMSPro documentation system (Box). The agency’s quality improvement officer received email notification after each electronically recorded intubation encounter. He then completed telephone follow up with the treating paramedic if any data points were missing in the record.

The ambulance service in this study is a 10-site EMS agency throughout two Midwestern states that had 230 paramedic and nonparamedic (eg, emergency medical technician–basic [EMT-Basic]) part-time or full-time employees working during the study period. The population of each service area ranges from less than 20,000 to more than 100,000 persons. For the 5-month study period, all patient encounters involving at least one intubation attempt were included in the analysis.

### Statistical Methods

We evaluated comparisons of features (both patient features and aspects of the EMS experience) between patients with successful intubation and those with unsuccessful intubation using the 2-sample t test, the χ^2^ test, and the Fisher exact test. Associations with successful intubation were evaluated using logistic regression and were summarized with odds ratios (ORs) and 95% CIs. We built a multivariable logistic regression model using a stepwise selection process with the p-value set to <0.05 for a feature to be included in the model. The predictive ability of the features in the multivariable model was summarized using the area under the receiver operating characteristic curve. We performed statistical analyses were performed using the SAS version 9.2 software package (SAS Institute Inc). All tests were 2-sided and p-values <0.05 were considered statistically significant.

## RESULTS

During the study period, which included 12,527 emergent ambulance responses, 150 patients (147 adults aged 18 to 94 years and 3 children aged <1 to 16 years) underwent 200 intubation attempts. Features of the last intubation attempt per patient are summarized in Table 1. Intubation was successful in most patients (n=113, 75%) but was unsuccessful in 25% (n=37). A comparison of features between patients with successful and unsuccessful intubation is shown in Table 2. Results of the univariate logistic regression models are summarized in Table 3. Smaller patient size by weight (p=0.03), a crew with two paramedics (“paramedic/paramedic”) (p=0.01), the first intubation attempt (p=0.02), small endotracheal tube size (p=0.02), and a view of some (p=0.002) or all of the glottis (p<0.001) were significantly associated with successful intubation by univariate analysis.

Results of the multivariable logistic regression model are summarized in Table 4. A paramedic/paramedic crew was more than three times as likely to achieve successful intubation compared with a crew of one paramedic and one EMT-Basic (“paramedic/EMT-Basic”) (OR, 3.30; p=0.03). A small endotracheal tube of 6, 6.5, or 7 inches was associated with a more than 4-fold increased likelihood of successful intubation compared with a large endotracheal tube (7.5 or 8 inches) (OR, 4.25; p=0.01). After adjustment for these features, our analysis showed that, compared with no or little view of the glottis, a partial (“some”) or complete (“entire”) view of the glottis was associated with a nearly 13-fold and a nearly 40-fold increased likelihood of successful intubation, respectively (OR, 12.98; p=0.001 and OR, 39.78; p<0.001). The area under the receiver operating characteristic curve for this model was 0.88, which indicates that the features in the model contained high predictive ability.

### Rapid Sequence Intubation

Of the 37 patients who underwent attempted rapid sequence intubation (RSI), 27 (73%) had successful outcomes and 10 (27%) had unsuccessful outcomes. An entire view of the glottis was associated with a more than 8-fold increased likelihood of successful RSI compared with no, little, or some view of the glottis (OR, 8.33; p=0.03). After adjustment for view of the glottis, no other feature was significantly associated with RSI success.

## DISCUSSION

Our 75% overall success rate per patient in one or more ETI attempts is similar to rates reported previously. However, many factors significantly affected successful placement. After controlling for these features, we found that the ability of paramedics to achieve at least “some” view of the glottis was the best predictor overall of the likelihood of a successful ETI. A successful ETI was achieved on the first attempt in 90 patients (80%), whereas the success rate for all ETI attempts was 56% (113/200).

### Frequency of Skill Usage

The need for the application of ETI skills was rare in this study population. The 150 patients requiring airway management in this sample represented only 0.01% of the 12,527 ambulance responses during the 5-month study period. Konrad et al.^8^ found that anesthesia residents achieved a 90% success rate after a mean of 57 ETI cases; 18% of the residents still required assistance even after 80 attempts. A second study of anesthesia residents found an 88.9% success rate after 27 cases,^9^ whereas another study evaluating “non-anesthesia trainees” defined a “good intubation” as requiring 47 prior attempts.^10^

In our study, 103 EMS providers attempted 200 total intubations (data on intubator were missing in 10 cases), which equated to 1.94 attempts per provider. The EMS agency employs 230 paramedics, which equates to 0.87 attempts per provider for the 5-month study period. There were no documented ETI attempts by 127 paramedics (55%). In the absence of clinical rotations, obtaining the number of cases or attempts recommended in the anesthesia-focused studies cited above would be unachievable for the average paramedic throughout a career.

Wang et al.^11^ suggested that 15 to 25 ETI attempts in different clinical settings may be needed to achieve a 90% rate of successful intubation. Despite such evidence, national paramedic educational requirements remain at five intubations per student.

### EMS Crew Configuration

Multivariable logistic regression showed that paramedic/paramedic crews were more than three times as likely to achieve successful intubation as paramedic/EMT-Basic crews (OR, 3.30; p=0.03). With medical guidelines limiting attempts to two per paramedic provider, paramedic/paramedic crews have twice as much ETI capacity (four attempts vs two attempts) as paramedic/EMT-Basic crews. However, in our study, paramedic/paramedic crews made 124 ETI attempts on 92 patients (1.35 attempts per patient), compared with 76 attempts on 58 patients (1.31 attempts per patient) by paramedic/EMT-Basic crews.

### Glottis View

Both univariate and multivariable logistic regression showed significantly higher success rates when “some” or “all” of the glottis was viewed by the intubator. In a previous multivariable analysis, the inability to view vocal cords was also significantly associated with unsuccessful intubation.^12^ Eliminating intubation attempts in patient populations with other important contributors to ETI failure (ie, larger patients requiring larger ET tubes) may increase the proportion of attempts in which full view of the glottis is obtained. External laryngeal maneuvers may assist in obtaining better glottis views.^13^,^14^ (13,14). The proper external laryngeal techniques aimed at increasing views of the glottis include external laryngeal manipulation and BURP (backwards upwards rightwards pressure), both of which have been shown to improve the view of the glottis.^15^ A 2006 report on bimanual manipulation indicated that it improved the view more than either cricoid pressure (CP) or BURP.^16^.

CP is a controversial topic without clear consensus, but it is currently indicated for intubations in the medical guidelines of this EMS. When done incorrectly, CP can cause airway obstruction.^17^,^18^ Retention of this skill has been shown to range from less than one month to more than three months.^19^,^20^ Neither CP nor BURP was studied in this dataset, and neither technique is taught at the study site.

### Patient Weight and Endotracheal Tube Size

By multivariable analysis, lower patient weight was a univariate predictor of ETI success, as was smaller tube size (6, 6.5, and 7 in). The success rate with tubes seven inches or smaller was 83%, vs 66% for tubes larger than seven inches, for an absolute difference of 17%. For the purposes of this discussion, we associated endotracheal tube size with patient size. Increased weight was shown to be a key factor in another multivariable logistic regression model with a larger sample size (>650 patients)^12^

## LIMITATIONS

This study has several limitations. Foremost is that the data collected on airway encounters were self-reported. The study team attempted to mitigate this effect by having direct telephone follow-up calls with the EMS crews if data elements were missing, but these calls could not be completed in all cases. Furthermore, although we attempted to follow up with the crews in a timely fashion (within several days of the ETI incident), an element of recall bias may remain. CP is a controversial topic and, as discussed, can be detrimental to ETI success. Our system does not practice this technique, but we did not query its use in our assessments. It is possible that paramedics could still use CP and it not be reflected. ETI also could be considered a rarely performed skill in the prehospital setting, which may lead to generalizability issues with these data.

## CONCLUSION

In this EMS cohort of prehospital airway management cases, successful ETI was best accomplished when a paramedic was partnered with another paramedic, when the intubator had at least some view of the glottis, and when the intubator elected to use a smaller endotracheal tube. In our EMS, each paramedic is teamed up with either another paramedic or with an EMT-Basic. Our results suggest that the makeup of the prehospital care team (paramedic/paramedic vs paramedic/EMT-Basic) may affect the success of intubation. We hypothesize that a paramedic who performs intubation with another paramedic has a skilled partner or coach to provide additional guidance not available from an EMT-Basic during this challenging scenario. Additional work on evaluating backup airways and examining new intubation techniques and tools, such as video laryngoscopy, may also help increase success rates in the future.

## Figures and Tables

**Table 1:** Patient characteristics (n=150) and features of the EMS experience with intubation.

Feature	Value^a^
Patient characteristics	
Age, y	58.0 (19.5)
Weight, kg (n=148)	83.5 (25.6)
Sex	
Male	104 (69)
Female	46 (31)
Height, feet	(n=139)
4 to 4 ½	1 (1)
4 ½ to 5	2 (1)
5 to 5 ½	49 (35)
5 ½ to 6	80 (58)
6 to 6 ½	7 (5)
Features of EMS experience	
Crew	
Paramedic/EMT-basic	58 (39)
Paramedic/paramedic	92 (61)
Intubator experience, y (n=131)	5.9 (0.6–33.3)
Intubator experience, y	(n=131)
<1	7 (5)
1 to 4	53 (40)
5 to 9	25 (19)
≥10	46 (35)
Intubation attempts	
1	112 (75)
2	29 (19)
3	6 (4)
4	3 (2)
Cervical collar	
No	116 (77)
Yes	34 (23)
Rapid sequence intubation	(n=149)
No	112 (75)
Yes	37 (25)
Endotracheal tube size, mm, internal diameter	(n=149)
6	3 (2)
6.5	3 (2)
7	82 (55)
7.5	53 (36)
8	8 (5)
Glottis view	(n=139)
Entire	95 (68)
Some	19 (14)
Little	12 (9)
None	13 (9)
Stylet	(n=147)
Gum bougie	103 (70)
Wire/satin slip	38 (26)
Other	6 (4)

*EMS*, emergency medical services; *EMT*, emergency medical technician

a Values are mean (SD), median (range), or No. of patients (%)

**Table 2:** Features of patients with and without successful intubation.

	Intubation group^a^		
		
Feature	Unsuccessful (n=37)	Successful (n=113)	p-value
Patient characteristics			
Age, y	52 (15–94)	62 (18–92)	0.12
Weight, kilograms (n=148)	79 (54–181)	79 (29–181)	0.03
Sex			0.07
Male	30 (81)	74 (65)	
Female	7 (19)	39 (35)	
Height, ft	(n=34)	(n=105)	0.05
4 to 5 ½	8 (24)	44 (42)	
5 ½ to 6 ½	26 (76)	61 (58)	
Features of EMS experience			
EMS crew			0.009
Paramedic/EMT-basic	21 (57)	37 (33)	
Paramedic/paramedic	16 (43)	76 (67)	
Intubator experience, y (n=131)	5.0 (0.8–30.7)	7.1 (0.6–33.3)	0.22
Intubator experience, y	(n=35)	(n=96)	0.61
<1	1 (3)	6 (6)	
1 to 4	16 (46)	37 (39)	
5 to 9	8 (23)	17 (18)	
≥10	10 (29)	36 (38)	
Intubation attempts			0.03
1	22 (59)	90 (80)	
2	13 (35)	16 (14)	
3	2 (5)	4 (4)	
4	0	3 (3)	
Intubation attempts			0.01
1	22 (59)	90 (80)	
>1	15 (41)	23 (20)	
Cervical collar			0.10
No	25 (68)	91 (81)	
Yes	12 (32)	22 (19)	
Rapid sequence intubation		(n=112)	0.72
No	27 (73)	85 (76)	
Yes	10 (27)	27 (24)	
Endotracheal tube size, mm, internal diameter	(n=36)		0.02
6, 6.5, or 7	15 (42)	73 (65)	
7.5 or 8	21 (58)	40 (35)	
Glottis view	(n=30)	(n=109)	<0.001
Entire	8 (27)	87 (80)	
Some	4 (13)	15 (14)	
Little	5 (17)	7 (6)	
None	13 (43)	0	
Stylet	(n=34)		0.44
Gum bougie	27 (79)	76 (67)	
Wire/satin slip	6 (18)	32 (28)	
Other	1 (3)	5 (4)	

*EMS*, emergency medical services; *EMT,* emergency medical technician

a Values are median (range) or No. of patients (%)

**Table 3:** Univariate associations with successful endotracheal intubation.

Feature	Odds ratio (95% CI)	p-value
Patient characteristics		
Age, per 10-y increase	1.16 (0.96–1.40)	0.13
Sex		
Male	1.00 (reference)	
Female	2.26 (0.91–5.61)	0.08
Weight, per 10-kilogram decrease (n=148)	1.17 (1.01–1.34)	0.03
Height, feet (n=139)		
4 to 5 ½	1.00 (reference)	
5 ½ to 6 ½	0.43 (0.18–1.03)	0.06
Features of EMS experience		
EMS crew		
Paramedic/EMT-basic	1.00 (reference)	
Paramedic/paramedic	2.70 (1.26–5.76)	0.01
Intubator experience, per 1-y increase (n=131)	1.04 (0.98–1.10)	0.22
Intubator experience, y (n=131)		
<5	1.00 (reference)	
5 to 9	0.84 (0.31–2.31)	0.74
≥10	1.42 (0.58–3.49)	0.44
Intubation attempts		
1	1.00 (reference)	
>1	0.38 (0.17–0.83)	0.02
Cervical collar		
No	1.00 (reference)	
Yes	0.50 (0.22–1.16)	0.11
Rapid sequence intubation (n=149)		
No	1.00 (reference)	
Yes	0.86 (0.37–2.00)	0.72
Endotracheal tube size, mm, internal diameter (n=149)		
6, 6.5, or 7	1.00 (reference)	
7.5 or 8	0.39 (0.18–0.84)	0.02
Glottis view (n=139)		
None or little	1.00 (reference)	
Some	9.64 (2.36–39.36)	0.002
Entire	27.96 (9.00–86.94)	<0.001
Stylet (n=147)		
Gum bougie	1.00 (reference)	
Wire/satin slip	1.90 (0.71–5.03)	0.20
Other	1.78 (0.20–15.90)	0.61

*EMS,* emergency medical services; *EMT,* emergency medical technician

**Table 4:** Multivariable associations with successful endotracheal intubation in patients treated by EMS staff.

Feature	Odds ratio (95% CI)	p-value
EMS crew		
Paramedic/EMT-basic	1.00 (reference)	
Paramedic/paramedic	3.30 (1.13–9.69)	0.03
Endotracheal tube size, mm, internal diameter		
7.5 or 8	1.00 (reference)	
6, 6 ½, or 7	4.25 (1.37–13.20)	.001
Glottis view		
None or little	1.00 (reference)	
Some	12.98 (2.69–62.54)	0.001
Entire	39.78 (10.81–146.35)	<0.001

*EMS,* emergency medical services; *EMT,* emergency medical technician

## References

[1] Tam RK, Maloney J, Gaboury I (2009). Review of endotracheal intubations by Ottawa advanced care paramedics in Canada. Prehosp Emerg Care.

[2] Wang HE, Mann NC, Mears G (2011). Out-of-hospital airway management in the United States. Resuscitation.

[3] Frascone RJ, Russi C, Lick C (2011). Comparison of prehospital insertion success rates and time to insertion between standard endotracheal intubation and a supraglottic airway. Resuscitation.

[4] Holmberg TJ, Bowman SM, Warner KJ (2011). The association between obesity and difficult prehospital tracheal intubation. Anesth Analg.

[5] Deakin CD, King P, Thompson F (2009). Prehospital advanced airway management by ambulance technicians and paramedics: is clinical practice sufficient to maintain skills?. Emerg Med J.

[6] Warner KJ, Carlbom D, Cooke CR (2010). Paramedic training for proficient prehospital endotracheal intubation. Prehosp Emerg Care.

[7] Wang HE, Domeier RM, Kupas DF (2004). Recommended guidelines for uniform reporting of data from out-of-hospital airway management: position statement of the National Association of EMS Physicians. Prehosp Emerg Care.

[8] Konrad C, Schupfer G, Wietlisbach M (1998). Learning manual skills in anesthesiology: is there a recommended number of cases for anesthetic procedures?. Anesth Analg.

[9] Charuluxananan S, Kyokong O, Somboonviboon W (2001). Learning manual skills in spinal anesthesia and orotracheal intubation: is there any recommended number of cases for anesthesia residency training program?. J Med Assoc Thai.

[10] Mulcaster JT, Mills J, Hung OR (2003). Laryngoscopic intubation: learning and performance. Anesthesiology.

[11] Wang HE, Seitz SR, Hostler D (2005). Defining the learning curve for paramedic student endotracheal intubation. Prehosp Emerg Care.

[12] Wang HE, Kupas DF, Paris PM (2003). Multivariate predictors of failed prehospital endotracheal intubation. Acad Emerg Med.

[13] Vanner RG, Clarke P, Moore WJ (1997). The effect of cricoid pressure and neck support on the view at laryngoscopy. Anaesthesia.

[14] Benumof JL, Cooper SD (1996). Quantitative improvement in laryngoscopic view by optimal external laryngeal manipulation. J Clin Anesth.

[15] Harris T, Ellis DY, Foster L (2010). Cricoid pressure and laryngeal manipulation in 402 pre-hospital emergency anaesthetics: essential safety measure or a hindrance to rapid safe intubation?. Resuscitation.

[16] Levitan RM, Kinkle WC, Levin WJ (2006). Laryngeal view during laryngoscopy: a randomized trial comparing cricoid pressure, backward-upward-rightward pressure, and bimanual laryngoscopy. Ann Emerg Med.

[17] Brimacombe JR, Berry AM (1997). Cricoid pressure. Can J Anaesth.

[18] Ho AM, Wong W, Ling E (2001). Airway difficulties caused by improperly applied cricoid pressure. J Emerg Med.

[19] Herman NL, Carter B, Van Decar TK (1996). Cricoid pressure: teaching the recommended level. Anesth Analg.

[20] Shimabukuro A, Kawatani M, Nagao N (2006). Masui.

